# Seroprevalence Trends and Molecular Epidemiology of Viral Hepatitis in Croatia

**DOI:** 10.3390/life13010224

**Published:** 2023-01-13

**Authors:** Tatjana Vilibic-Cavlek, Snjezana Zidovec-Lepej, Thomas Ferenc, Vladimir Savic, Tatjana Nemeth-Blazic, Mateja Vujica Ferenc, Maja Bogdanic, Maja Vilibic, Bojana Simunov, Natasa Janev-Holcer, Pavle Jelicic, Dominik Ljubas, Tian Kosar, Maja Ilic, Jasmina Kucinar, Ljubo Barbic, Vladimir Stevanovic, Anna Mrzljak

**Affiliations:** 1 Department of Virology, Croatian Institute of Public Health, 10000 Zagreb, Croatia; 2 School of Medicine, University of Zagreb, 10000 Zagreb, Croatia; 3 Department of Immunological and Molecular Diagnostics, University Hospital for Infectious Diseases “Dr. Fran Mihaljevic”, 10000 Zagreb, Croatia; 4 Clinical Department of Diagnostic and Interventional Radiology, Merkur University Hospital, 10000 Zagreb, Croatia; 5 Poultry Center, Croatian Veterinary Institute, 10000 Zagreb, Croatia; 6 Department of Communicable Disease Epidemiology, Croatian Institute of Public Health, 10000 Zagreb, Croatia; 7 Department of Obstetrics and Gynecology, University Hospital Centre Zagreb, 10000 Zagreb, Croatia; 8 Department of Psychiatry, Sestre Milosrdnice University Hospital Center, 10000 Zagreb, Croatia; 9 School of Medicine, Catholic University of Croatia, 10000 Zagreb, Croatia; 10 Department of Nephrology, Merkur University Hospital, 10000 Zagreb, Croatia; 11 Environmental Health Department, Croatian Institute of Public Health, 10000 Zagreb, Croatia; 12 Department of Social Medicine and Epidemiology, Faculty of Medicine, University of Rijeka, 51000 Rijeka, Croatia; 13 Medical Centre Zagreb City Centre, 10000 Zagreb, Croatia; 14 Department of Microbiology, Istria County Institute of Public Health, 52100 Pula, Croatia; 15 Department of Microbiology and Infectious Diseases with Clinic, Faculty of Veterinary Medicine, University of Zagreb, 10000 Zagreb, Croatia; 16 Department of Gastroenterology and Hepatology, University Hospital Center Zagreb, 10000 Zagreb, Croatia

**Keywords:** Hepatotropic viruses (A–E), seroprevalence, molecular epidemiology, Croatia

## Abstract

Viral hepatitis is a significant cause of morbidity and mortality worldwide. In Croatia, hepatitis B virus (HBV) and hepatitis C virus (HCV) are widely distributed, especially in some high-risk groups such as people who inject drugs (PWID), prisoners, and highly promiscuous groups. The seroprevalence of HBV ranges from 7.0% in the general population to 38.8% in PWID, depending on the region. The seroprevalence of HCV is highest among PWID (29–75.5%) as compared to 0.9% in the general population. Analyzing the distribution of HCV genotypes, no substantial changes in the molecular epidemiology of the two most frequent HCV genotypes (1 and 3) in the past 20 years were observed. However, the predominance of subtype 1b compared to subtype 1a as detected in 1996–2005 was not confirmed in 2008–2015. Hepatitis A virus (HAV) incidence was high in the past with a decreasing trend since the 2000s, except for an outbreak in 2017–2018 as part of the large European outbreak, which was mainly among men who have sex with men. Hepatitis E virus (HEV) is an emerging virus detected for the first time in Croatia in 2012. The seroprevalence of HEV is high among hemodialysis patients (27.9%) and liver transplant recipients (19.3–24.4%). In addition, higher seroprevalence rates were observed in animal-related professions (e.g., veterinarians, 15.2%; hunters, 14.9%). All detected HEV strains belonged to genotype 3.

## 1. Introduction

Viral hepatitis is a major global healthcare concern and a significant cause of morbidity and mortality. The main hepatotropic viral pathogens are hepatitis A virus (HAV), hepatitis B virus (HBV), hepatitis C virus (HCV), hepatitis D virus (HDV), and hepatitis E virus (HEV). These viruses may cause various forms of the disease (e.g., asymptomatic, acute, chronic, fulminant) and have the potential to emerge as focal outbreaks or initiate epidemic spread. HAV is a member of the *Picornaviridae* family, genus *Hepatovirus*. It has a worldwide distribution, annually causing 1.4 to 1.5 million hepatitis cases [1,2,3]. The virus is primarily transmitted via the fecal–oral route, and due to its close association with poor living standards and hygiene conditions, there is a distinction in the proportion of HAV-infected individuals in different populations and geographic regions [2,3]. In developed Scandinavian countries, the reported HAV seroprevalence was 13%, whereas, in African and Asian countries, the rate has risen to 100% [1]. Although an infected individual is typically asymptomatic, adults are more likely to experience the disease’s severe forms, such as fulminant hepatitis [4]. HBV is a member of the *Hepadnaviridae* family, genus *Orthohepadnavirus*. It is also a globally distributed hepatotropic virus with 350 million infected individuals every year [5,6,7]. There is an estimation that 5% of HBV infections progress into a chronic form of the disease with an increased risk of developing liver failure, cirrhosis, or hepatocellular carcinoma (HCC) [8,9]. HBV is highly endemic in the Sub-Saharan region of Africa, whereas the burden of this infection is less impactful in North American, Latin American, and western European countries [9,10]. According to the annual 2019 Global Burden of Disease report, in Western Europe, the highest HBsAg seroprevalence was detected in Greece (1.8%), in Central Europe in Bulgaria (2.4%), and in Eastern Europe in Ukraine (1.3%). The virus is commonly transmitted through sexual contact, exposure to needles contaminated with infected blood, or during the perinatal period [9]. Therefore, the population groups at the highest risk of getting infected with HBV are men who have sex with men (MSM), promiscuous heterosexual individuals, and people who inject drugs (PWID) [5]. HDV is a member of the *Deltaviridae* family, genus *Deltavirus*. It has similar transmission routes to HBV; however, it is significantly less frequent compared to HBV. HDV causes one of the most severe forms of hepatitis in people with chronic HBV infections [11]. In the worldwide general population, HDV’s seroprevalence varies greatly from 0.0% to 8.03% [12,13]. HCV belongs to the *Flaviviridae* family, genus *Hepacivirus*. According to some estimations, 2–3% of the global population is infected with HCV, and individuals infected with HCV mostly develop a chronic, lifelong infection that causes long-term liver damage, which can include cirrhosis or HCC. Consequently, it results in a high mortality rate of 350,000–400,000 deaths every year [14,15]. HCV infection has a worldwide prevalence of 0.4% to 5.2% with lower seroprevalence rates in northern and western European countries (0.9%) compared to eastern European countries (3.3%) [16]. This bloodborne virus can be transmitted through transfusion, hemodialysis, inadequately sterilized medical equipment, organ transplantation, intravenous drug use, and sexual contact [17]. HCV has seven genotypes, each of whose distribution is determined by the mode of transmission, and genotypes 1 (subtype 1b) and 3 are the most common in Europe [17,18]. HEV belongs to the *Hepeviridae* family within the genus *Orthohepevirus*. It is accountable for an estimated 20 million infections each year [19,20,21]. HEV is considered to be the most common cause of viral hepatitis, and it has eight different genotypes, with genotypes 1 and 2 being restricted to humans [22]. HEV infection usually presents as a self-limiting illness with low mortality (1–2%), but immunocompromised patients can develop more severe forms of the disease. The disease is prevalent in the developing countries of eastern and southern Asia, which hold more than 60% of all documented cases [23]. In these areas, the main route of transmission is fecal–oral through contaminated drinking water supplies, with human-specific genotypes HEV-1 and HEV-2 being the predominant cause of infection [19]. On the other hand, in developed parts of the world (e.g., Europe), the source of infection is often the consumption of insufficiently thermally processed meat from an infected animal. On these occasions, genotypes with zoonotic potential (HEV-3 and HEV-4) are the leading causes of the disease. The reported prevalence of HEV in Europe is between 0.6% and 52.5% [23].

As mentioned above, certain hepatitis virus seroprevalence rates in Europe are still varying between different population groups and geographic regions. This review aims to analyze the published data on viral hepatitis in Croatia and summarize the epidemiological characteristics and molecular epidemiology of major hepatitis viruses A–E in different population groups in order to interpret these results in the European context.

## 2. General Population

**Figure 1:**
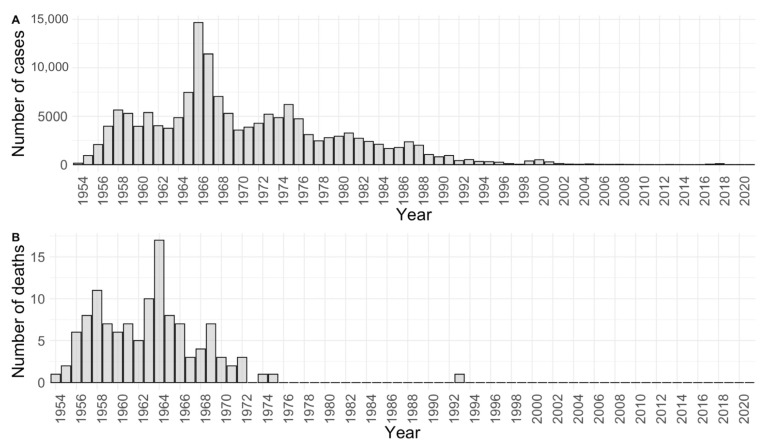
Hepatitis A in Croatia—historical data (1954–2020): number of reported cases (**A**) and deaths (**B**) in the National Notifiable Diseases Surveillance System.

Hepatitis A has been monitored in Croatia since 1954. The HAV was a significant cause of morbidity and mortality from the 1950s to the 1970s, with 5000 to 14,000 cases reported annually (Figure 1). A continuous decrease in the incidence of hepatitis A was observed, with an average incidence rate of 100/100,000 in the 1950s and less than 1/100,000 in the last decade, due to socioeconomic development and improved hygiene standards [24]. A considerable increase in incidence was observed in 2017 and 2018 when 96 and 47 cases of hepatitis A, respectively, were recorded as part of the epidemic that spread from Europe to Croatia [25]. The last increase in HAV incidence was noted in 2022, with 202 cases reported (preliminary data from the Reference Center for Epidemiology of the Croatian Ministry of Health, Croatian Institute of Public Health) reflecting outbreaks from several EU and EEA countries as well as the United Kingdom which started in December 2021 [26]. A significant proportion of cases in these outbreaks were associated with contact among MSM.

The seroprevalence of viral hepatitis A–E in the Croatian general population differs greatly. Only two published studies analyzed the seroprevalence of HAV. In a study conducted during 2008–2009, the overall anti-HAV seroprevalence was 41.6%. Similarly, seroprevalence was 40.5% in a study conducted in 2010–2011. Both studies showed that HAV seropositivity in Croatia was age-dependent with a marked increase in seroprevalence with age, whereas it was not affected by gender or by rural or urban residence [1,15]. Literature data on the molecular epidemiology of HAV in the general population are exceptionally limited. Only one study on an imported case reported an HAV subtype IB infection in a 20-year-old woman with diabetes type I from Croatia hospitalized with hepatitis A upon returning from Burkina Faso [27].

During a two-year period (2010–2011), a large seroprevalence study on HBV was conducted in patients from four Croatian counties who underwent routine check-ups. The overall prevalence of HBsAg and anti-HBc or anti-HBs was 0.7 and 7.0%, respectively, whereas the prevalence of isolated anti-HBs was 24.4% (postvaccinal immunity). Anti-HBc seropositivity increased significantly with age, rising from 1.7% in the 20–29-year age group to 15.8% in individuals above 60 years. In addition, residents of rural areas showed significantly higher anti-HBc seropositivity than residents of urban areas (10.7 vs. 6.1%). Gender was not associated with HBV seroprevalence [15].

Very few studies analyzed the presence of HDV in Croatia. In one general hospital, 19% of the 100 patients with chronic liver disease (CLD) who tested HBV positive also showed positive markers of HDV infection. Most of them were PWID, and HDV infection was linked to severe clinical manifestations of CLD (89.5% had cirrhosis and chronic active hepatitis, whereas 19.5% had chronic persistent hepatitis) [28].

**Figure 2:**
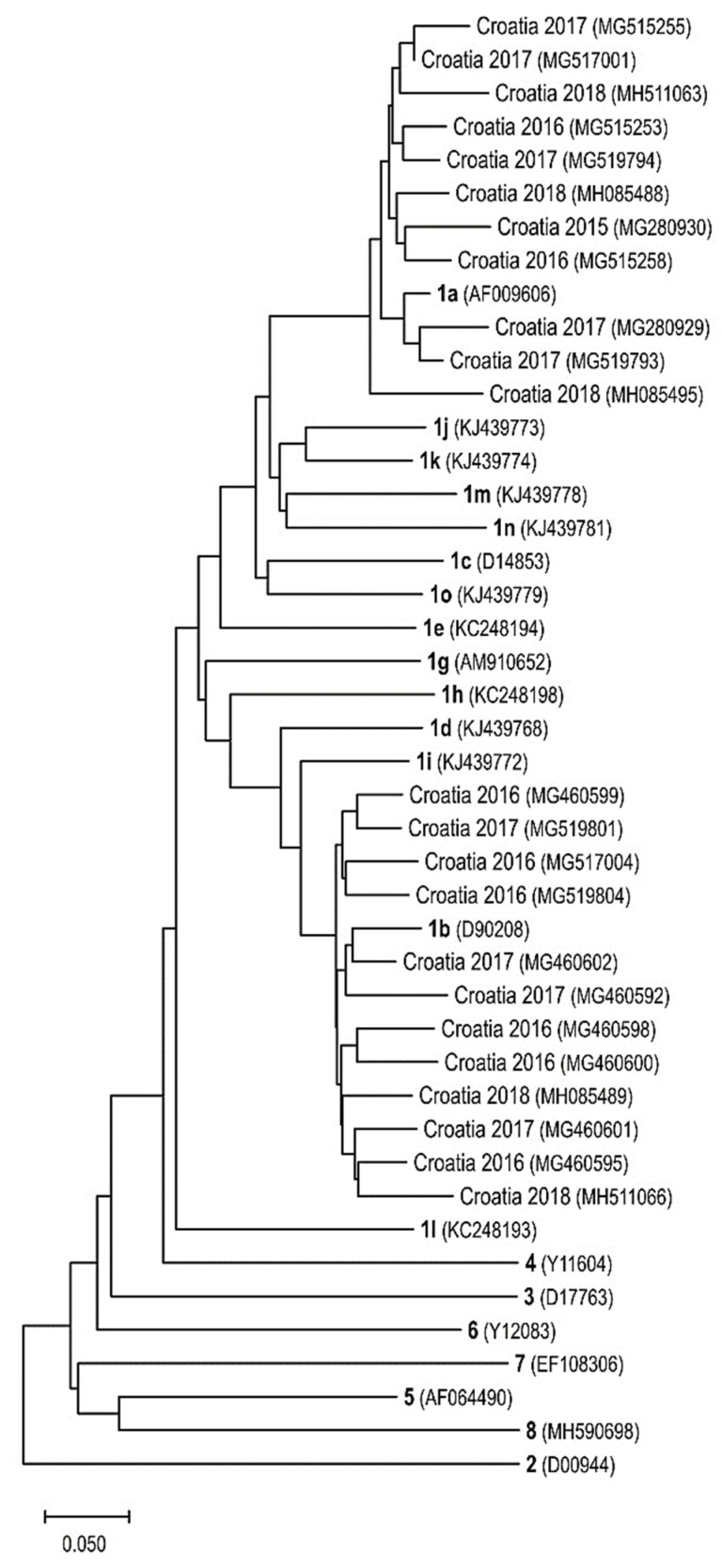
Phylogenetic tree of selected human hepatitis C virus (HCV) isolates from Croatia and genotype 1–8 representative strains as well as subtype 1a-1o representative strains (in bold). Designations include GenBank accession numbers (in parentheses) and detection years for Croatian strains. A maximum likelihood phylogenetic analysis was conducted, and an evolutionary analysis was performed by using MEGA11 [32].

Several studies analyzed the prevalence of anti-HCV and HCV RNA in the Croatian general population. Anti-HCV antibodies were detected in 0.9% of individuals in 2010–2011 [15]. HCV genotypes were analyzed in patients with chronic hepatitis C in the period 1996–2005 and 2008–2015 [29,30]. There were no substantial changes in the molecular epidemiology of the two most frequent HCV genotypes in Croatia in the past 20 years. During 2008–2015, HCV genotype 1 was the most prevalent in Croatian patients (56.6%), followed by genotypes 3 (37.3%), 4 (4.2%), and 2 (1.8%). The central–northwestern and eastern regions had the highest prevalence of genotype 1 (64.7 and 65.0%, respectively), whereas the central–southern Adriatic region had the lowest prevalence (45.8%). The central–southern Adriatic region had the highest prevalence of genotype 3 infections (49.1%) compared to Eastern Croatia (17.5%) [30]. However, the significant predominance of subtype 1b as compared to subtype 1a (37.4 vs. 13.1%) that was observed in 1996–2005 was not confirmed in 2008–2015 (54.0 vs. 46.0%) [29,30]. It is important to note that these differences were, at least partly, due to different methods used for genotyping. In the earlier study, a first-generation line-probe INNO-LiPA HCV 1.0 molecular assay that targeted the 5′ noncoding region of the HCV genome was used. Some authors showed that INNO-LiPA HCV 1.0 failed to correctly identify HCV subtype 1a in 29.5% and subtype 1b in 8.7% of samples, whereas second-generation INNO-LiPA HCV 2.0 assay used in a more recent study that targets 5′non-coding and core regions of HCV genome correctly classified 97.5% of subtype 1a and 96.2% of subtype 1b strains [31]. The presented data showed a high degree of HCV molecular diversity (Figure 2) on a national and regional level, indicating the need for continuous surveillance of HCV molecular epidemiology.

A prospective pilot study on HEV conducted in 2014–2015 showed an overall HEV IgG seropositivity rate of 5.6% with a significant increase in seroprevalence with age, rising from 1.8 to 2.3% in individuals younger than 40 years to 11.3% in individuals older than 50 years. Residents of suburban and rural areas were more often seropositive than residents of urban areas (14.5 vs. 2.5%). In addition, an increasing prevalence of HEV IgG antibodies (12.1%) was observed in participants living in families with more than four household members compared to 1.8% in those living with fewer than two members [33].

**Figure 3:**
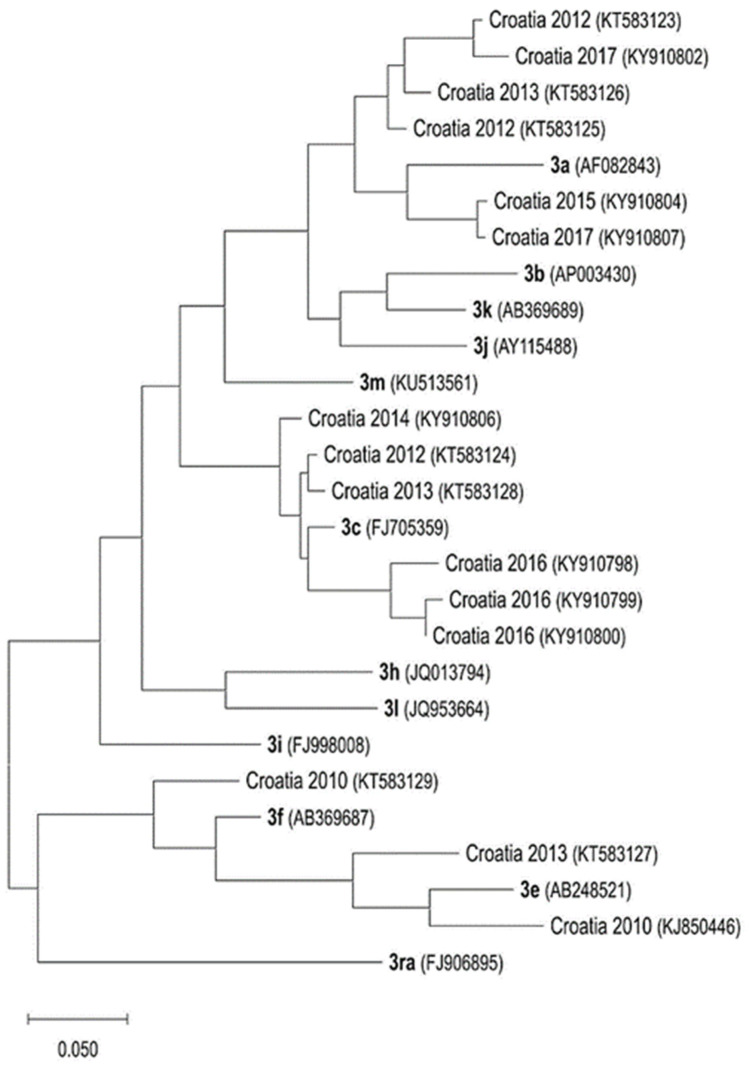
Phylogenetic tree of HEV human isolates from Croatia and genotype 3 subtype reference strains (in bold) according to Smith et al. [35]. Designations include GenBank accession numbers (in parentheses) and detection years for Croatian strains. A maximum likelihood phylogenetic analysis was conducted, and an evolutionary analysis was performed by using MEGA11 [32].

HEV genotypes were analyzed in serum samples collected from anti-HEV-positive patients (2012–2017) at the Referent Centre for Viral Hepatitis at the University Hospital for Infectious Diseases “Dr. Fran Mihaljevic”, Zagreb, as well as from archive samples of patients with hepatic lesions of unknown origin. All detected strains were identified as members of *Orthohepevirus* A species, genotype 3. In addition, all strains were genetically closely related even though they clustered into two general subgroups and four subtypes (3a, 3c, 3e, and 3f) (Figure 3) [34].

## 3. People Who Inject Drugs

**Figure 4:**
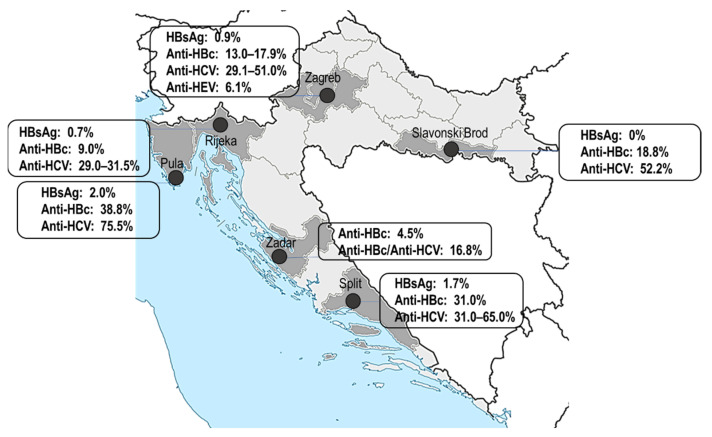
Prevalence of viral hepatitis markers in PWID in Croatia.

After the introduction of HCV testing in 1993, PWID represents a group with the highest risk for HCV infection. One seroprevalence study was conducted among current and former PWID registered at the Center for Prevention and Outpatient Treatment of Addiction in Zadar, located at the Croatian littoral, from 2002 to 2005. HBV monoinfection was found in 4.5% PWIDs, whereas 16.8% of them showed serologic evidence of HBV–HCV coinfection (Figure 4) [36]. From 2005 to 2007, PWID from seven Croatian cities were tested for the presence of HCV antibodies. The majority of patients (69.8%) reported multiple potential exposures to HCV, including sharing injecting equipment (97.1%), risky sexual behavior (75.0%), and traveling abroad previously (56.3%). The overall HCV seroprevalence rate was 51.3% and increased with an increasing number of risk behaviors. The most significant risk factor associated with HCV seropositivity was needle-sharing frequency. The seroprevalence rate increased from 27.3% in PWID who shared needles occasionally to 100% in PWID who always shared needles [37]. In 2007, HBV prevalence and risk factors were analyzed in PWID from the three largest Croatian cities (Zagreb, Split, and Rijeka). The target group included PWID in prison settings as well as PWID who participated in certain harm reduction programs in communities. The prevalence rates of HBsAg, anti-HBc, and anti-HCV were 0.5–1.7%, 9.0–31.0%, and 29.0–65.0%, respectively [38] (Figure 4).

From 2012 to 2014, PWID from geographically distant regions (three counties in the Croatian mainland and one county located on the Adriatic Coast) were tested. The overall prevalence rates of HBsAg, anti-HBc, and anti-HCV were 0.9, 22.8, and 55.8%, respectively. There were significant regional differences in both anti-HBc (17.9–38.8%) and anti-HCV prevalence rates (49.1–75.5%). According to the data of the National register of persons treated for psychoactive drug abuse, the highest rate of addiction per 100,000 inhabitants was noted in Istria County (507.1) in which the highest HCV seropositivity was reported. In Zagreb, Zagreb County, and Brod-Posavina County, addiction rates were 304, 116.5, and 117.7, respectively. Pula is a tourist place located on the Adriatic Coast with a larger movement of people and goods which could be a possible reason for the higher HCV prevalence rate than in continental regions. Anti-HBc prevalence increased gradually with age from 21.3 to 64.3%, starting with the 30–39 age group. A sharp increase in anti-HCV seroprevalence was observed from 24.6% in PWID less than 30 years to 59.6% in the 30–39 age group. Thereafter, there was a steady increase to 92.9% in IDUs above 50. Sharing injection equipment correlated strongly with HBV and HCV seropositivity. Significantly higher seroprevalence rates were found in PWID who reported sharing syringes, needles, or both either frequently or occasionally (anti-HBc 34.7/23.5% vs. 14.9%; anti-HCV 87.8/63.5 vs. 8.7%) (unpublished data of the Croatian Institute of Public Health).

In 2014–2015, PWID from Zagreb, Split, and Rijeka were tested for HCV. The prevalence rates of HCV antibodies were 29.1% in Zagreb, 38.3% in Split, and 31.5% in Rijeka [39]. In addition, in PWID from Split and Rijeka, risk factors were analyzed. The results showed that both Split and Rijeka PWID aged 35 years or older as well as those who were injecting drugs for more than 10 years had higher odds of anti-HCV positivity. Among PWID from Split, anti-HCV seroprevalence positively correlated with a history of imprisonment, whereas negatively correlated with having none or one sexual partner in the previous year [40].

## 4. Healthcare Workers

Data on the prevalence of hepatitis viruses in Croatian healthcare workers (HCWs) are very scarce. Occupational exposures have been monitored in one university hospital from Zagreb during the period from January 2002 to December 2011. Among the exposed HCWs, 59.4% had protective anti-HB titers. HBV seropositivity was 1.6% and HCV seropositivity was 2.2% in tested HCWs [41]. The preliminary results of the HEV seroprevalence testing in a small cohort of HCWs tested in 2014 and 2017 showed seropositivity of 2.7% and 2.0%, respectively [33,42].

## 5. Persons with High-Risk Sexual Behavior

Several studies were conducted on individuals with high-risk sexual behavior in Croatia, including MSM, homo- or bisexual persons, commercial sex workers (CSW), persons with multiple sexual partners, and persons with sexually transmitted diseases (STDs) (Table 1).

In 2006, a prevalence study on STDs was conducted among the MSM population at the HIV VCT Centre (University Hospital for Infectious Diseases, Zagreb). The HAV, HBV (anti-HBc), and HCV seropositivity rates were 14.2%, 9.2%, and 3.0%, respectively [43].

The distribution of HAV genotypes among MSM was analyzed during the European epidemics that disproportionally affected men (particularly MSM, in part HIV-infected) in the period 2016–2018 (2016: 12,430 cases; 2017: 26,145 cases; 2018: 15,680 cases reported to the ECDC) [45]. In 22 EU and EEA countries, in the period between June 2016 and September 2018, a total of 4475 outbreak-confirmed cases have been confirmed. Outbreak-confirmed cases were defined as EU or EEA residents with laboratory-confirmed HAV genotype IA and a sequence with ≥99.3% homology to one of the three HAV genotype IA outbreak strains (VRD_521_2016; RIVM-HAV16-090; and V16-25801) based on overlapping fragments in the VP1-2a region. Increased numbers of hepatitis A cases during 2017 and 2018 have also been reported in Croatia (2017: 47 cases; 2018: 96 cases). Molecular analysis of Croatian MSM patients (including HIV-infected ones) with hepatitis A that was diagnosed between February 2017 and August 2018 showed the presence of VRD_521_2016 (associated with traveling to the UK and Spain, *n* = 17 patients) and RIVM-HAV16-090 (Europride) strains (*n* = 18 patients) [46]. Subsequently, the incidence of hepatitis A in Croatia (2019: nine cases; 2020: five cases; 2021: five cases) was similar to 2016 (*n* = 5 cases) and prior to that period. However, in 2022, a distinct cluster of HAV genotype IA-positive MSM (HIV-infected and users of pre-exposure prophylaxis) had also been characterized in Croatia [47].

A seroepidemiological study of HBV in groups with high-risk behaviors in which the vast majority of patients were among clients of VCT centers was conducted from 2011 to 2013 in 11 Croatian counties. The study included MSM, homo- and bisexual persons, CSW and clients of CSW, and persons with multiple sexual partners. The overall prevalence of HBsAg and anti-HBc was 2.6% and 12.1%, respectively. Anti-HBc seroprevalence differed among genders with a higher prevalence among men (13.8% vs. 5.7%). In addition, anti-HBc positivity increased progressively with age from 3.3% in individuals less than 30 years old to 25.0% in those aged 40–49 years and remained stable thereafter, whereas the prevalence of HBsAg was highest in the 40–49-year age group (7.4%) compared with 1.3–3.7% in other age groups. Furthermore, individuals with a history of STDs were more often seropositive than participants who did not report STDs (HBsAg 8.2% vs. 1.0%; anti-HBc 32.4% vs. 6.4%) [5].

From 2003 to 2006, the seroprevalence, risk factors, and HCV genotypes in groups with high-risk sexual behavior from seven Croatian cities were analyzed. Anti-HCV prevalence rates varied from 2.9% to 8.5% with an overall prevalence of 4.6%. Among sexually transmitted disease markers, a higher HCV seroprevalence rate was found in individuals with either a prior HBV infection (10.5 vs. 3.8%) or gonorrhea (13.2 vs. 4.2%). No other factors reflecting risky sexual behavior, such as sexual orientation and the number of sexual partners, were associated with HCV seroprevalence. HCV RNA was detected in 73.1% of anti-HCV-positive patients. Interestingly, three of the seronegative individuals (2.1%) were also found to be HCV-RNA positive (“window period”). Genotype 1 was most commonly detected (55.6%), whereas the most prevalent subtypes were 1a (38.9%) and 3a (38.9%) [44]. More recent studies on the prevalence of viral hepatitis in persons with high-risk sexual behavior are lacking.

There are only preliminary data on HEV infection in individuals with high-risk sexual behavior, showing an IgG seroprevalence rate of 1.9% (unpublished data of the Croatian Institute of Public Health).

## 6. Prison Population

Incarcerated persons comprise about 0.4% of the Croatian population, and 25–30% of them abuse drugs. A large survey was carried out between December 2005 and December 2007 and included 3348 adults, 144 juveniles, and 259 members of the correctional staff from 20 Croatian prisons. The study population represented 32.9% of the total Croatian adult inmates, 72.9% of the total juvenile prisoner population, and 10.9% of the total prison staff. The most prevalent risk group for adults was PWID (24.3%), followed by alcoholics (11.9%) and highly promiscuous persons (8.2%). In contrast, among juveniles, the most prevalent was the highly promiscuous group (22.9%). The alcoholics and PWID comprised 13.2% and 12.5% of the tested population, respectively. In total, 25.9% of prisoners were positive for some viral hepatitis markers. The prevalence of HBV infection was highest in PWID and highly promiscuous groups (26.2% and 20.4%, respectively). HCV infection was most commonly detected in PWID (52.2%) [48]. HDV infections in the prison population were found to be very low (0.15%) [49]. From 2007 to 2009, a total of 190 Croatian male prisoners from four Croatian prisons were tested for HBV and HCV markers. About half of them reported more than one potential risk behavior: 34.8% used injected drugs, 62.5% had multiple sexual partners, 13.0% engaged in male-to-male sex, 22.6% engaged in paid sex, 75.6% engaged in unprotected sex with casual partners, and 41.7% had tattoos or piercings. Two participants (1.1%) tested positive for HBsAg, 7.9% for anti-HBc, and 10.6% for anti-HCV. The HCV seropositivity rate was correlated to IDU and history of tattoos [50]. Given that the majority of HCV-positive prisoners indicated additional potential exposures to HCV, such as sharing injection equipment or engaging in high-risk sexual behavior, it was unclear whether tattooing represented a real risk factor for HCV transmission in this population. The presented results indicated that HBV and HCV were widely distributed in the Croatian prison population. Because a substantial proportion of prisoners who are released back into society continue to spread these infections at a high rate, correctional initiatives should be included in prevention and control strategies.

## 7. Pregnant Women

**Figure 5:**
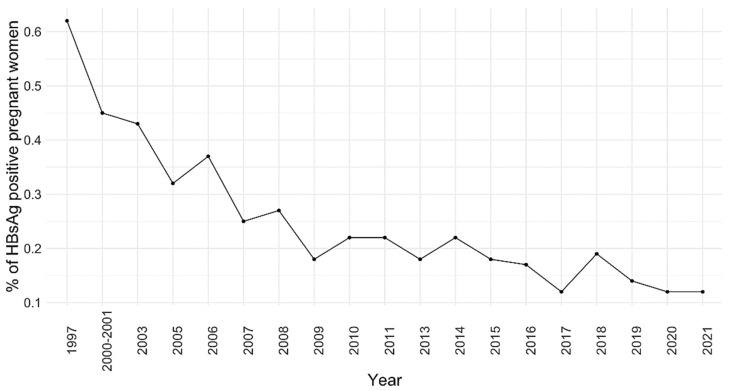
Prevalence of HBsAg in Croatian maternity hospitals (1997–2021).

The viral hepatitis prevalence data among Croatian pregnant women is scarce. One of the first studies on this matter was conducted in 1999 in the Split region (Croatian littoral) to determine the strategy for the prenatal screening of HBV infection. The authors’ study population included 400 pregnant women with a mean age of 27.8 ± 5.5 (17–43 years). Results showed that 7.5% of women were seropositive for past or current HBV infection, whereas 0.75% of patients were chronic carriers. The main routes of infection were through transfusion or occupational or sexual exposure, and five women had previously documented hepatitis of unclear etiology [51]. Another study conducted in the same region in 2008 examined neonatal outcomes in pregnant women with a positive history of drug abuse. Among other data, it was found that 16.0% of women with a known addiction were carriers of HBV and 49.0% of them of HCV, whereas 11.0% were simultaneously coinfected with HBV and HCV. In comparison, the control group of non-drug-using childbearing women had seropositivity for HBV, HCV, and coinfection of HBV and HCV of 2.0, 0.04, and 0.04%, respectively [52]. Data from Croatian maternity hospitals showed a decline in the HBsAg prevalence rate in pregnant women from 0.65% in 1997 to around 0.2% since 2007 (Figure 5).

Only a few studies referred to the seroprevalence of HCV in the pregnant population. In the review from 2015, two regional studies were mentioned from Zagreb County (2003–2006) and Istria County (2011–2012) in which the detected HCV seroprevalence rates were 0.5 and 1.3%, respectively [17,53].

In the most recent study conducted in continental Croatian regions (2022), 118 pregnant women were tested for the presence of HEV IgG antibodies, and only two of them were positive (1.7%) [19].

To this date, there have been no conducted studies on HAV seroprevalence in the pregnant population of Croatia.

## 8. Hemodialysis Patients

Patients receiving maintenance hemodialysis are at high risk for bloodborne viruses; therefore, HBV and HCV serological status is monitored routinely. Historical studies, before mandatory testing of blood products and successful erythropoietin-stimulating agent therapies, reported high prevalence rates. In 1992, up to 44.0% of hemodialysis patients showed anti-HCV antibodies [54]. A similar anti-HCV seropositivity rate (38.0%) was noted in 1994 [55] with a reported 10.0% HBsAg prevalence. A declining trend in HCV seroprevalence was observed thereafter to 23.0% in 2001 [56]. More recent regional data showed much lower and more stable seroprevalence rates in Croatian hemodialysis patients (2.3%–3.2%) [57,58]. Confirmatory to the above-mentioned trends are low seroprevalence rates for anti-HCV in patients enlisted for kidney transplantation.

There is less published data on HBV seroprevalence, although a similar declining trend was observed, from the beginning of mandatory blood product testing to recent times [59].

HEV seroprevalence in hemodialysis patients has been evaluated in a recent multicenter study, and a high seropositivity rate of 27.9% was found. There was significant geographical variation, with rates ranging from 5.2 to 43.4%. Factors associated with HEV IgG seropositivity were age > 60 years (OR 8.17; 95% CI = 1.08–62.14), living in the continental Croatian regions (OR 2.58; 95% CI = 1.55–4.30), and previous transfusion of blood products (OR 1.66; 95% = CI 1.01–2.73) [60]. Given the high seroprevalence rate in certain regions, HEV screening should be considered in the differential diagnosis of hepatitis, especially in immunocompromised patients.

## 9. Solid Organ Transplant Recipients

Published data on viral hepatitis seroprevalence in solid organ transplant recipients (SOTRs) in Croatia are scarce, even though serological status for HBV and HCV is a prerequisite for enlisting on the deceased donor list and part of the routine work-up for all organ recipients. A large 10-year study (2006–2016) analyzed serological profiles of 1308 SOTRs, 452 kidney transplant recipients (KTRs), and 856 liver transplant recipients (LTRs). The prevalence of HBV and HCV–HBV coinfection in SOTRs was low, as well as the prevalence of anti-HCV in KTRs at the time of transplant. Overall, 1.5% of KTRs and 5.4% of LTRs were HBsAg positive, whereas 2.2% of KTRs and 3.4% of LTRs were only anti-HBc positive. The prevalence of successful pretransplant HBV vaccine immunity was higher in the KTRs than in the LTRs (36.9 vs. 5.5%). Anti-HCV antibodies were detected in 2.2% of KTRs and 15.7% of LTRs, whereas 1.1% of kidney and 0.2% of liver recipients were both HBsAg and anti-HCV positive [59]. The serological status of SOTR is not monitored routinely but is tested upon clinical indication.

Given the more recent scientific interest in HEV, there are more data on HEV seroprevalence. In 2019, a study on 242 LTRs showed an HEV IgG seroprevalence rate of 24.4%, whereas no HEV RNA was detected. Older age, female gender, living in a rural area or on a farm, well water usage, and septic tank-related factors were associated with HEV seropositivity [22,61]. In a very recently published study (2022), seropositivity was high in LTRs (19.3%), whereas in KTRs, the seroprevalence was similar to the Croatian general population (6.9 and 7.1%, respectively) [19]. These results conflict with the high seroprevalence in hemodialysis patients, reflecting the lower average age of KTRs and geographical variations. Larger confirmatory studies are needed in both populations.

## 10. Blood Donors

**Figure 6:**
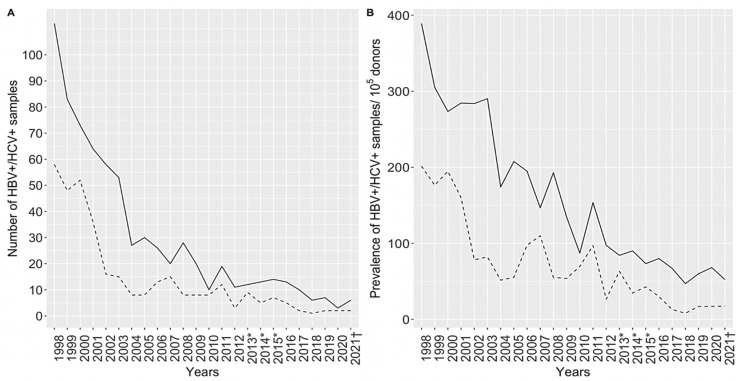
Prevalence of HBV (line) and HCV (dashes) infection in voluntary blood donors (1998–2021): number of positive samples (**A**), prevalence/10^5^ donors (**B**); serology results, serology + NAAT *, NAAT †.

Screening of voluntary blood donors (VBD) for hepatitis viruses was continuously performed at the Croatian Institute of Transfusion Medicine. The prevalence of HBV and HCV per 100,000 first-time blood donors in Croatia from 1998 to 2021 is presented in Figure 6. Continuous decreases in both HBV and HCV markers were observed, from 0.39 to 0.05% for HBV and from 0.2 to 0.02% for HCV [62].

The anti-HBc prevalence trends were assessed over a 14-year period (2004–2017). They showed a decrease from 5.24% in 2004 to 2.56% in 2013 and to 1.32% in 2017. HBsAg and HBV DNA were not detected [63].

Anti-HEV antibodies were analyzed in 2014. Of the samples tested, 21.5% were reactive to total HEV antibodies with a significant association between age and HEV seroprevalence. The highest seropositivity was observed in the 40–49- and 50–59-year age groups (30.7 and 27.8%, respectively), compared to 1.8–17% in other groups. In addition, significant regional differences in the seroprevalence rates were found [64].

The prevalence of HBV and HCV among blood donors in Croatia showed a continuous decline over time. However, the seroprevalence of HEV infection is high, especially in individuals above 50 years.

## 11. Forestry Workers, Hunters, Veterinarians

Only one published study analyzed HEV seroprevalence in exposed individuals. The study was conducted between 2016 and 2017 at the Croatian Institute of Public Health. The exposed population included hunters, veterinary professionals, and forest workers, whereas the control group was the general population. The study showed a significant difference in HEV seroprevalence among the exposed population; veterinarians had an HEV seroprevalence rate of 15.2%, hunters had 14.9%, forestry workers had 6.5%, and the general population had 7.1%. HEV seropositivity significantly rose with age, from 2.9% in people under 30 to 23.5% in people over 60. The risk factors for acquiring HEV infection were also analyzed. It was shown that food preferences (consumption of game meat, offal, and pork products), environmental and housing factors (type of drinking water supply, type of water drainage or sewer, waste disposal, domestic animals, etc.), and eating habits (consumption of game meat, offal, and pork products) were not linked to HEV seropositivity. However, those who claimed to own pets were more likely to be seropositive than those who did not (12.5 vs. 7.0%) [19]. Although the presented study showed that professional exposure to animals seems to be a risk factor for HEV seropositivity, further studies on a large sample are needed to confirm this observation.

## 12. Conclusions

A declining trend of HAV incidence was observed in Croatia, except for an outbreak among MSM in 2017–2018 as part of the European outbreak and a distinct cluster of HAV genotype IA-positive HIV-infected MSM detected in 2022 [25,46]. The notification rate of hepatitis A in the EU and EEA also declined between 1997 and 2011 from 10.0 to 2.5 per 100,000 population. However, several travel-related, community-wide, and foodborne HAV outbreaks were reported in the past decade in the general population as well as in high-risk groups, including PWID [65].

The prevalence of HBsAg in the Croatian general population (0.7%) is in line with the European average. Over the past ten years, European trends have suggested a general decrease in the prevalence of acute and chronic HBV infection, but disease patterns remained diverse and dynamic, with significant variations in prevalence between studies [66,67]. The average national prevalence of chronic hepatitis B is 0.9%, ranging from 0.01% in the United Kingdom and Ireland to 10.32% in Kyrgyzstan. Eastern Europe continues to have a higher HBsAg prevalence, particularly in Moldova (7.38%), Romania (5.61%), and Bulgaria (3.92%) [67].

Hepatitis C is widely distributed among Croatian PWID (29–75.5%). A recently published study that included 17 European countries showed similar or even higher HCV seroprevalence rates, especially among incarcerated PWID, from 50.0 to 60.0% (United Kingdom, Austria) and up to 91.5% in Latvia [68]. A large majority of HCV patients are infected with genotype 1 or 3, and very few with 2 or 4 [69]. Similarly, HCV genotype 1 was the most prevalent in Croatian patients (56.6%), followed by genotypes 3 (37.3%), 4 (4.2%), and 2 (1.8%) [30].

Like in other parts of Europe, HEV seroprevalence in Croatia varies greatly depending on the population studied (1.7% in pregnant women, 24.4% in LTRs, and up to 43.3% in hemodialysis patients). Seroprevalence rates in other European countries are reported to be 2%–9.7% in Albania, up to 9.7% in Greece, 5.9–17.1% in Romania, up to 20.9% in Bulgaria, and 15% in Serbia [61]. All Croatian HEV strains belonged to genotype 3, which is the most frequent causative genotype in HEV infections in developed countries [70,71,72,73].

Favorable epidemiological trends regarding viral hepatitis in Croatia, particularly in the general population, are encouraging. Extensive national efforts in monitoring viral hepatitis seroprevalence and molecular epidemiology helped identify the critical targets for future interventions in the prevention, diagnosis, and treatment of viral hepatitis. The high prevalence of HCV and HBV infections in vulnerable patient groups, particularly IDUs, prisoners, and highly promiscuous groups, will require additional targeted interventions in the future. Following the recovery from the COVID-19 pandemic, refocusing on monitoring trends in viral hepatitis, particularly in emerging HEV infection among hemodialysis patients, liver transplant recipients, and persons in animal-related professions, as well as HAV infection among MSM, must become an integral part of the national viral hepatitis surveillance.

## Figures and Tables

**Table 1:** Prevalence of viral hepatitis markers in groups with high-risk sexual behavior in Croatia.

Population Group	Viral Hepatitis Marker	Year(s) Tested	N Tested	N (%) Positive	95%CI	Reference
Clients from voluntary counseling and testing centers ^a^	HBsAg	2011–2013	443	11 (2.6)	1.3–4.5	[5]
	Anti-HBc	2011–2013	443	54 (12.1)	9.1–15.5	
MSM population	Anti-HAV	2006	360	(14.2)	9.1–19.8	
	HBsAg	2006	360	2 (0.5)	0.1–1.9	[43]
	Anti-HBc	2006	360	33 (9.2)	5.2–13.1	
	Anti-HCV	2006	360	11 (3.0)	1.1–5.3	
		2003–2006	205	6 (2.9)	0.5–5.2	[44]
	Anti-HEV	2014–2015 2018–2019	53	1 (1.9)	0.1–10.1	[33], CIPH, unpublished data
Persons with multiple sexual partners	Anti-HCV	2003–2006	378	24 (6.3)	3.9–8.8	[44]
Persons with a history of STDs		2003–2006	199	17 (8.5)	4.7–12.4	[44]

MSM = men who have sex with men; CSW = commercial sex workers; STDs = sexually transmitted diseases; CIPH = Croatian Institute of Public Health; ^a^ = includes MSM, bisexual persons, CSW or clients of CSW, and highly promiscuous persons.

## Data Availability

Not applicable.
